# Effect of Electrode Thickness on Quality Factor of Ring Electrode QCM Sensor

**DOI:** 10.3390/s22145159

**Published:** 2022-07-09

**Authors:** Zhenfang Wei, Jianguo Hu, Yuanyuan Li, Jing Chen

**Affiliations:** 1 School of Integrated Circuits, Beijing National Research Center for Information Science and Technology (BNRist), Tsinghua University, Beijing 100084, China; zhenfangwei@home.hpu.edu.cn (Z.W.); liyy2020@mail.tsinghua.edu.cn (Y.L.); chenjing152713@tsinghua.edu.cn (J.C.); 2 School of Physics & Electronic Information Engineering, Henan Polytechnic University, Jiaozuo 454001, China

**Keywords:** QCM, ring electrode, quality factor, electrode thickness

## Abstract

As a key type of sensor, the quartz crystal microbalance (QCM) has been widely used in many research areas. Recently, the ring electrode QCM sensor (R-QCM) with more uniform mass sensitivity has been reported. However, the quality factor (Q-factor) of the R-QCM has still not been studied, especially regarding the effect of electrode thickness on the Q-factor. Considering that the Q-factor is one of crucial parameter to the QCM and it is closely related to the output frequency stability of the QCM, we study the effect of different electrode thicknesses on the Q-factor of the R-QCM in this paper. On the other hand, we clarify the relationship between the electrode thickness and the Q-factor of the R-QCM. The measurement results show that the average Q-factor increases with increases in the thickness of ring electrodes generally; however, the resonance frequency of the QCM resonator decreases with increases in the thickness. The low half-bandwidth (2Γ < 1630 Hz) of the R-QCM shows that the frequency performance is good. Additionally, the R-QCM has a higher Q-factor (Q > 6000), which indicates that it has a higher frequency stability and can be applied in many research areas.

## 1. Introduction

The quartz crystal microbalance (QCM) is a balance for the detection of small signals in many research areas. It consists of two metal electrodes and a thin quartz crystal plate. The QCM has been widely used due to its simple structure, low cost and ability to detect small mass changes in the order of nanograms [1,2,3,4,5,6,7].

As a key parameter for the QCM, the quality (Q) factor hints that the frequency is stable in its core part, the resonator. Generally speaking, the QCM with an overly low Q-factor will lead to low reproductivity and high errors in measurement results in applications. Recently, QCMs with asymmetrical electrodes have become a focus due to the fact that they improve the sensor’s performance [8,9]. For example, a modified electrode quartz crystal resonator with a higher point mass sensitivity and Q-factor than the conventional QCM was proposed [10,11]; A. Richardson et al. presented a type of QCM with a ring electrode design to achieve the desired uniform mass sensitivity distribution [12]; Zhu et al. proposed the optimization of the electrode size and the mass ratio of the electrode to the plate to realize the uniform mass sensitivity distribution of the ring electrode QCM [13]; X.H. Huang et al. studied the mass sensitivity distribution of the ring electrode QCM [14]; Y. Yao et al. found that a QCM with an asymmetric sensitive electrode structure improves humidity sensitivity [15]. On the other hand, D.Z. Zhang et al. developed QCM humidity sensors based on graphene film [16,17,18]. 

Our previous research found that the mass sensitivity of the ring electrode QCM is higher than that of the conventional QCM with a symmetrical electrode [19]. These abovementioned studies showed that QCMs with asymmetrical electrode structures improve mass sensitivity greatly. However, the effect of a ring electrode on the Q-factor has not been reported in these studies, especially with regard to the effect of the thickness of ring electrodes on the Q-factor. Therefore, it is necessary to clarify the relationship between the Q-factor and the thickness of ring electrode QCM.

In this work, the effect of electrode thickness on the quality factor of the R-QCM was proposed for the first time. After that, we provided the quality factor of R-QCMs with different electrode thicknesses. According to the different thicknesses of electrodes, we divided the R-QCMs into four groups (A, B, C and D). Group A, B, C and D were four R-QCMs with 500 Å, 1000 Å, 1500 Å and 2000 Å electrode thicknesses, respectively. The experimental results showed that the average Q-factor increases with increases in the thickness of ring electrodes generally; however, the resonance frequency of the QCM resonator decreases with increases in thickness. Additionally, the R-QCM has a higher Q-factor (Q > 6000), which indicates that it has a higher frequency stability and can be applied in many research areas.

## 2. Ring Electrode QCM and Q-Factor Calculation

**Figure 1:**
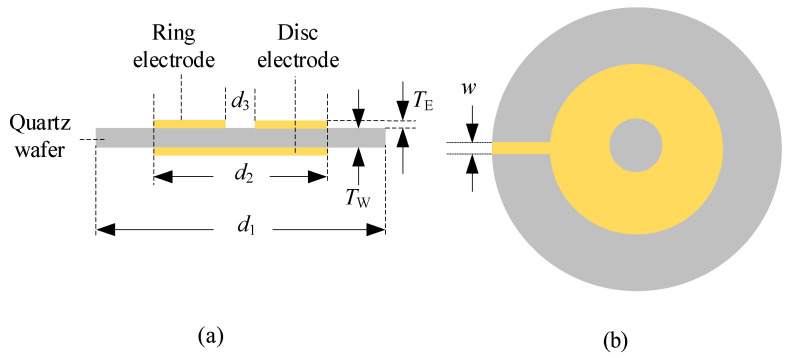
Structure of R-QCM. (**a**) Side view; (**b**) top view.

The core part of the R-QCM (Wintron, Zhengzhou, China) is an AT-cut quartz crystal resonator with the standard resonance frequency of 10 MHz. This structure of the AT-cut quartz crystal resonator is shown in Figure 1. The ring electrode thicknesses (*T*_E_) of group A, B, C, and D were 500 Å, 1000 Å, 1500 Å and 2000 Å, respectively. It should be noted that the thickness of the disc electrode was equal to the ring electrode thickness, and the electrode was made up of Au. *d*_1_ (*d*_1_ = 8.7 mm) was the quartz wafer diameter. *d*_2_ (*d*_2_ = 5.1 mm) was the disc electrode diameter and also was the outer diameter of the ring electrode. *d*_3_ (*d*_3_ = 2 mm) was the inner diameter of the ring electrode. *T*_W_ (*T*_W_ = 0.167 mm). W (W = 0.5 mm) was the width of the electrode contact pad.

**Figure 2:**
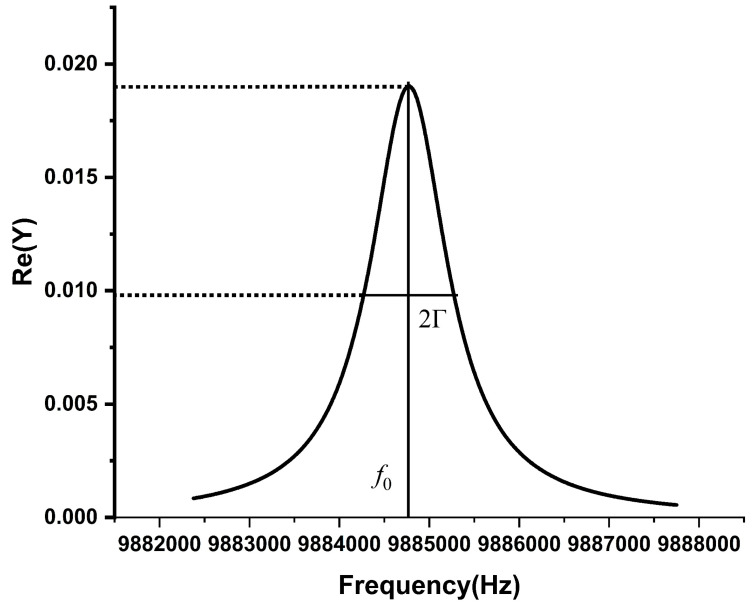
Schematic diagram of the resonance frequency and half-bandwidth.

It was developed based on the piezoelectric effect of quartz crystal material, and then, the German researcher Sauerbrey proposed the relationship between the change in resonance frequency and the small mass change in the electrode surface and described this relationship by an equation. This famous equation can be expressed as follows [20]:

(1) $$\Delta f=-\frac{2f_{0}^{2}}{\sqrt{\mu_{q}\rho_{q}}}\frac{\Delta m}{A},$$

where ∆*f* is the change in resonance frequency due to the change in electrode interfacial mass loading (∆*m*); *f*_0_ is the resonance frequency; *A* is the active electrode area; *μ_q_* (*μ_q_* = 2.947 × 10^11^ g/cm·s^2^) and *ρ_q_* (*ρ_q_* = 2.643 g/cm^3^) are the shear modulus of the AT-cut quartz crystal and the density of quartz, respectively. The negative sign in this equation hints that the change in resonance frequency is always opposite to the mass change in electrode surface. Namely, if the mass loading increases, the resonance frequency will be decreasing, and vice versa. The Q-factor is defined as the sharpness that describes the resonance frequency, and it determines the minimum frequency resolution [21]. On the other hand, the stability of resonance frequency is closely related with the Q-factor of the resonator. This relationship between the Q-factor and the stability of resonance frequency can be expressed by the fact that the higher Q-factor leads to more stability in the resonance frequency. In this study, the Q-factor of the R-QCM resonator was calculated by the method of actual measurement. According to the definition of the Q-factor, it can be expressed as [22]

(2) $$Q=\frac{f_{0}}{2\Gamma },$$

where *f*_0_ is the fundamental resonance frequency; 2Γ is half-bandwidth. These parameters are shown as Figure 2. The Q-factor describes the sharpness of the resonance frequency. This equation shows that the Q-factor is decided by resonance frequency and half-bandwidth. On the other hand, it also shows that when the resonance frequency is constant, the narrower the half-band width is and the higher the Q factor is, the better the frequency characteristic of the resonant cavity is.

Considering that the QCM resonator is widely used in applications, it is valuable to clearly confirm the Q-factor of the QCM resonator. In addition, the high Q-factor of the QCM resonator leads to the high reliability of its measurement. 

**Figure 3:**
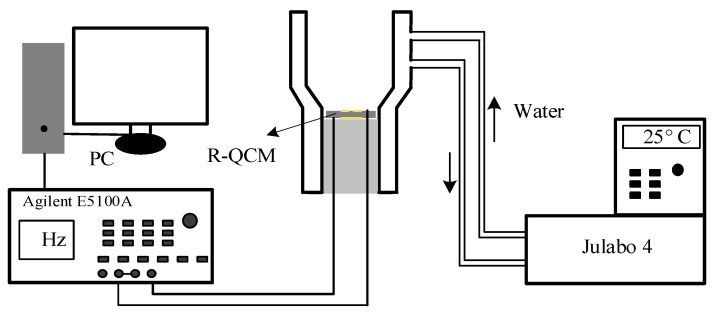
Measurements of resonance frequency and half-bandwidth.

The Q-factor of the R-QCM cannot be measured directly; however, it can be calculated and obtained using Equation (2). According to Equation (2), we designed the actual measurement experiment. First and foremost, the resonance frequency needed to be measured. Second, a suitable cell for the R-QCM had to be designed. Last, the temperature of the measurement needed to be controlled at 25 °C so as not to cause frequency deviation. Based on the above factors, we designed an experimental system. Figure 3 shows the experimental instruments used to measure resonance frequency and half-bandwidth. In the experimental systems, we used the network analyzer Agilent E5100A (Agilent technologies, Santa Clara, CA, USA) to measure the admittance spectrum near the resonance frequency. Considering the effect of the temperature frequency, the environmental temperature had to be kept at room temperature, 25 °C, and this was achieved using the Julabo 4 (Julabo GmbH, Seelbach, Germany) temperature controller. Then, the four groups of R-QCMs with different electrode thicknesses were measured using this system. Before starting the measurement, the ultrasonic cleaning of the R-QCM wafer was carried out. This process can clean small, attached impurities on electrode surfaces and further improve the accuracy of the measurement results. Then, we put the R-QCM into the cell. We set the frequency scanning range through the control software, so as to obtain the measurement data. After that, the resonance frequency and half-bandwidth of the R-QCM could be obtained by analyzing the measurement data.

## 3. Results

After measuring the resonance frequencies, the Q-factors of all groups were calculated using Equation (2). The relationship between the Q-factors of R-QCMs and the electrode thicknesses were obtained, and on the other hand, the effects of electrode thicknesses on Q-factors were also analyzed. These results contributed to fixing the low repeatability of the QCM sensors in the experiment.

The results of the Q-factors of R-QCMs are shown as in Table 1. In Table 1, *f*_0_ is the resonance frequency; $\bar{f_{0}}$ represents the average resonance frequency of each group; σ is the standard deviation of each group; 2Γ and $\bar{2}$Γ are the half-bandwidth of each R-QCM and the average half-bandwidth of each group; Q and $\;\bar{Q}\;$ are the quality factors of each QCM and the average quality factor of each group, respectively. It can be seen from this table that the maximum standard deviation of average resonance frequency in all groups is 1583.1 Hz. This result not only shows that the measured system is reliable, but it also shows that the resonance frequencies of the samples with the same thickness are in good agreement. The max average half-bandwidth in four groups is 1560 Hz and indicates that all the measured samples have good resonance frequency selectivity. In addition, all the R-QCMs have a high Q-factor (Q > 6000), and the average Q-factor of the four groups is 6418.3, 10,125.4, 9338.9 and 9500.3. 

**Figure 4:**
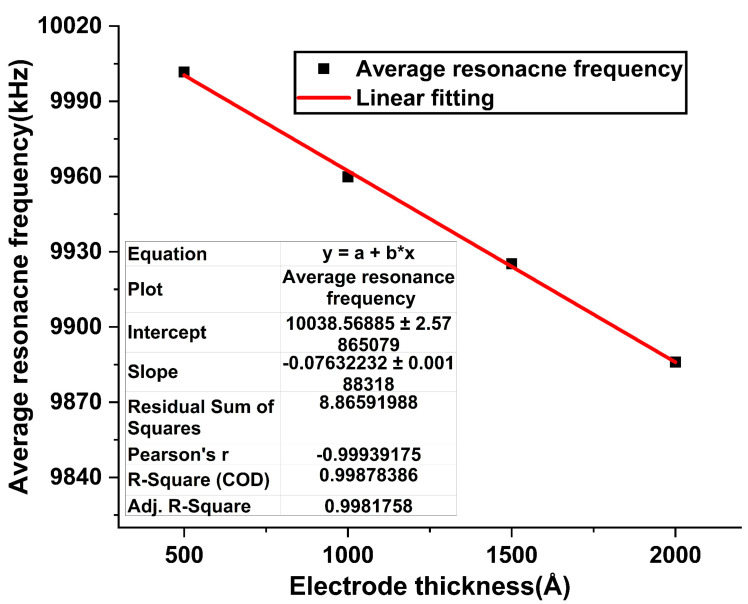
The relationship between resonance frequency of R-QCM and electrode thickness.

In order to know the relationship between the resonance frequency and the electrode thickness, we analyzed the measured results of all R-QCMs, and this relationship is shown in Figure 4. Adj. R-Square of fitting line is 0.998 and indicates that the fitting result and linearity are pretty good. It also shows that the decrease in the resonance frequency of the R-QCM increases with the electrode thickness.

## 4. Discussion

The Q-factor is a crucial parameter to R-QCM sensors. In our previous studies, the mass sensitivity distribution of the ring electrode R-QCM was more uniform than that of the conventional R-QCM, and its maximum mass sensitivity was closely related to the electrode thickness. However, the relationship between the Q-factor and the electrode thickness of the R-QCM is still unknown. Our research results show that the Q-factor varies with the change in electrode thickness, and the Q-factor increases with the increase in electrode thickness generally. On the other hand, the results also show that when a certain value of electrode thickness is obtained, there is a maximum value of the Q-factor. In the next step, we can find this maximum value of the Q-factor through theoretical calculations and measurements of more electrode thicknesses of R-QCMs.

## 5. Conclusions

In this paper, our focus was on the relationship between the Q-factor of R-QCMs and the electrode thickness. In order to illustrate this relationship, we measured the resonance frequency of R-QCMs. Firstly, according to the different thicknesses of electrodes, we divided the R-QCMs into four groups (A, B, C and D). Group A, B, C and D were four R-QCMs with 500 Å, 1000 Å, 1500 Å and 2000 Å electrode thicknesses, respectively. Then, we set up a measurement system to obtain the resonance frequency and half-bandwidth. The experimental results showed that the average Q-factor increases with increases in the thickness of ring electrodes generally; however, the resonance frequency of the R-QCM resonator decreases with increases in thickness. Additionally, the R-QCM has a higher Q-factor (Q > 6000), which indicates that it has higher frequency stability and can be applied in many research areas.

## Figures and Tables

**Table 1:** The resonance frequencies and Q-factors of R-QCMs.

	R-QCM (500 Å)	R-QCM (1000 Å)	R-QCM (1500 Å)	R-QCM (2000 Å)
*f*_0_ (Hz)	10,002,312	9,957,697.7	9,927,435	9,886,752.5
	9,999,564.1	9,959,473.8	9,924,772.1	9,885,251.8
	10,003,812	9,961,503.2	9,923,568.4	9,887,065.4
	10,000,901	9,960,540.3	9,925,232.5	9,884,773.1
$\bar{f_{0}}$ (Hz)	10,001,647.3	9,959,803.8	9,925,252.0	9,885,960.7
σ (Hz)	1583.1	1412.0	1399.2	969.6
2Γ (Hz)	1488.3	1030	1142.9	975.5
	1546.3	993.9	1171.5	1062.5
	1574.6	1093.3	952.1	1151.3
	1630.8	851.2	1015.4	991
$\bar{2}$Γ (Hz)	1560	992.1	1088.8	1045.1
Q	6720.6	9667.7	8686.2	10,135.1
	6466.8	10,020.6	8471.8	9303.8
	6353.2	9111.4	10,422.8	8587.7
	6132.5	11,701.8	9774.7	9974.5
$\bar{Q}$	6418.3	10,125.4	9338.9	9500.3

## Data Availability

Not applicable.
